# Early-life stress affects drug abuse susceptibility in adolescent rat model independently of depression vulnerability

**DOI:** 10.1038/s41598-020-70242-4

**Published:** 2020-08-07

**Authors:** Renata L. Alves, Pedro Oliveira, Igor M. Lopes, Camila C. Portugal, Cecília J. Alves, Fernando Barbosa, Teresa Summavielle, Ana Magalhães

**Affiliations:** 1 Addiction Biology, Instituto de Investigação e Inovação em Saúde (i3S) e Instituto de Biologia Molecular e Celular (IBMC), Universidade do Porto, 4200-135 Porto, Portugal; 2 Laboratory of Neuropsychophysiology, Faculty of Psychology and Education Sciences, University of Porto, 4200-135 Porto, Portugal; 3 Estudos de Populações, Instituto de Ciências Biomédicas Abel Salazar, Universidade do Porto, 4050-313 Porto, Portugal; 4 Glial Cell Biology, Instituto de Investigação e Inovação em Saúde (i3S) e Instituto de Biologia Molecular e Celular (IBMC), Universidade do Porto, 4200-135 Porto, Portugal; 5 Neuro and Skeletal Circuits, Instituto de Investigação e Inovação em Saúde (i3S) e Instituto Nacional de Engenharia Biomédica (INEB), Universidade Do Porto, 4200-135 Porto, Portugal; 6 Departamento de Ciências Funcionais, Escola Superior de Saúde (ESS), Politécnico do Porto, 4200-072 Porto, Portugal; 7 Behavioural Sciences, Instituto de Ciências Biomédicas Abel Salazar, Universidade do Porto, 4050-313 Porto, Portugal

**Keywords:** Developmental biology, Psychology

## Abstract

The development of substance abuse problems occurs due to a diverse combination of risk factors. Among these risks, studies have reported depression and early-life stress as of importance. These two factors often occur simultaneously, however, there is a lack of understanding of how their combined effect may impact vulnerability to drug abuse in adolescence. The present study used rats with different vulnerability to depression (Wistar and Wistar-Kyoto) to investigate the impact of maternal separation (MS) on emotional state and drug addiction vulnerability during the adolescence period. Mothers and their litters were subjected to MS (180 min/day) from postnatal day 2 to 14. The offspring emotional state was assessed by observing their exploratory behavior. Drug abuse vulnerability was assessed through conditioning to cocaine. MS impacted the emotional state in both strains. Wistar responded with increased exploration, while Wistar-Kyoto increased anxiety-like behaviours. Despite the different coping strategies displayed by the two strains when challenged with the behavioural tests, drug conditioning was equally impacted by MS in both strains. Early-life stress appears to affect drug abuse vulnerability in adolescence independently of a depression background, suggesting emotional state as the main driving risk factor.

## Introduction

The development of substance abuse problems occurs due to an almost unpredictable combination of risk factors. Among the common risk factors, mental disorder and emotional stress are constantly cited as important^1,2^. Among the main mental disorders, depression is one of the most common in adolescents, inspiring great concern due to its acute and long-lasting consequences^3^.

Depression in adolescence affects the physical, emotional and social development and can persist and recur into adulthood^4,5^. Addiction and depression can be described as having a bidirectional relationship, in which individuals that use drugs for recreational purposes are more likely to develop depression, while individuals that suffer from depression are more likely to develop addiction due to the consumption of drugs (or alcohol) to cope with the depressive symptoms^6,7^. Of note, depression and drug abuse comorbidity is highly prevalent in adolescence^8^.

Similarly to depression, emotional stress and its impact on one’s physical and affective condition may lead to the development of addiction. Furthermore, it is known that early life stress, such as children carelessness and mistreatment, can also induce anxiety and depression in adulthood^9^. This issue becomes especially relevant given the likelihood of the co-occurrence of these risk factors during early life. There is increased awareness about stressful emotional events that children go through, such as abuse (e.g. sexual, physical), negligence (physical and emotional) and common family and parental stress (poverty, displacement, hospitalization, mass immigration) that result in restricted access to quality early life care^10,11^. Besides, when drug abuse is installed, these co-occurring factors raise the risk of treatment failure, poorer treatment response, and earlier relapse^12^, which contributes to the lack of an effective drug abuse therapy^13^, making drug addiction treatment more challenging. Therefore, it is crucial to better understand if early-life stress impacts depression-prone children differently with respect to drug abuse.

In the present study, we hypothesized that maternal separation (MS) leads to a higher vulnerability to drug addiction and that this effect will be stronger for a depression-prone strain due to its altered emotional arousal. We aimed to investigate the impact of early-life stress, via MS, in promoting vulnerability to drug addiction in adolescent rats with different predisposition to depression. For that, we used Wistar-Kyoto (Kyoto) rats, a well-validated animal model of depression, recognized for displaying higher predisposition to anxiety and depression-like behaviours^14–17^, and the Wistar rat as a control strain.

## Results

### Elevated plus maze (EPM)

**Figure 1:**
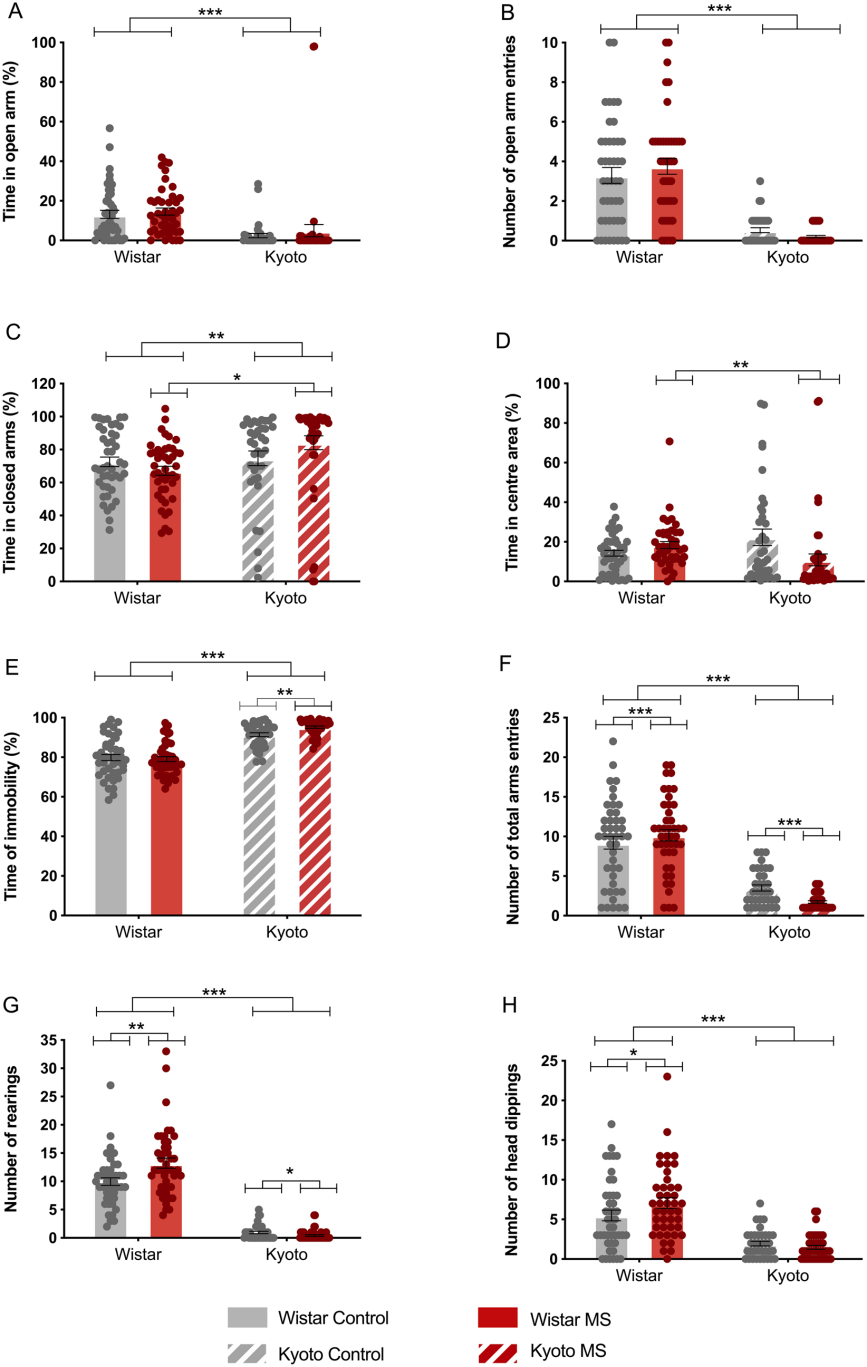
Effect of MS on the adolescent offspring anxiety assessed in the EPM. (**A**) time in open arms (%); (**B**) number of open arms entries; (**C**) time in closed arms (%); (**D**) time in centre area (%); (**E**) time immobile (%); (**F**) total entries; (**G**) number of rearing; (**H**) number of head dipping. Data were analysed by two-way ANOVA (2 × 2 factorial design) with Bonferroni comparisons except for: (**B**) Data were analysed by logistic regression. (**A**), (**C**), (**D**), (**E**) data were arcsine transformed. (**F**), (**G**), (**H**) data were square-root transformed. *n* = 44 except for Kyoto control (*n* = 36). Results are expressed as mean ± SEM. *p < 0.05, **p < 0.01, ***p < 0.001.

We first investigated if the protocol of MS used (daily separation from Post Natal Day—PND 2 to 14, 180 min/day) affected anxiety-like behaviour in both Wistar and Kyoto adolescent rats. To do so, we used the EPM test. Our results showed that the Wistar strain spent longer time in the open arms (*F* (1,164) = 48.9, *p* < 0.001) than the Kyoto (Fig. 1A) and entered the open arms more frequently (strain × MS: *Wald χ*^2^ = 3.91*, p* = 0.048; strain: *Wald χ*^2^ = 31.9*, p* < 0.001; MS: *Wald χ*^2^ = 4.11*, p* = 0.043, Fig. 1B). However, for both these measures, no significant differences could be attributed to MS. In accordance, the Kyoto rats spent more time in the closed arms (strain: *F*(1,164) = 10.3, *p* = 0.002, Fig. 1C) than the Wistar. We also found a strain × MS interaction (with *F*(1,164) = 7.29, *p* = 0.008 due to MS Kyoto spending more time in the closed arms than the MS Wistar (*p* = 0.044). On the other hand, MS Wistar spent more time in the centre area (*p* = 0.008) (Fig. 1D). In addition, the Kyoto rat presented higher time of immobility (strain × MS: *Wald χ*^2^ = 6.72*, p* = 0.010*;* strain: *Wald χ*^2^ = 148*, p* < 0.001) when compared with the Wistar (Fig. 1E). Immobility was also significantly increased when comparing MS Kyoto with control Kyoto (*p* = 0.004). These data are further **s**trengthened by a higher number of total arm entries by Wistar rats, which reflects the level of activity, and was substantially reduced in Kyoto rats (strain × MS: *Wald χ*^2^ = 9.99*, p* = 0.002*;* strain: *Wald χ*^2^ = 164, *p* < 0.001, Fig. 1F). Of note, the total number of arm entries is differently affected by MS, since it was increased in MS Wistar when compared with control Wistar (*p* < 0.001), and decreased in MS Kyoto (*p* < 0.001) when compared with control Kyoto. In harmony with the previous measures, exploration evaluation, represented by the number of rearings (strain: strain × MS: *F* (1,164) = 13.5, *p* < 0.001; strain: *F* (1,164) = 656, *p* < 0.001) and of head dippings (strain: strain × MS: *F* (1,164) = 5.28, *p* = 0.023; strain: *F* (1,164) = 76.8, *p* < 0.001) was lower in Kyoto rats, indicating that Kyoto performed less exploratory behaviours in the apparatus than Wistar animals (Fig. 1G,H). Again, MS seems to further increase exploration in MS Wistar (*p* = 0.002, Fig. 1G and *p* = 0.023, Fig. 1H) and decrease it in MS Kyoto (*p* = 0.037, Fig. 1G). Altogether, the results obtained in the EPM seem to indicate that, besides the strain related differences, MS promoted exploratory behaviours in Wistar rats, while downregulating it in Kyoto. Also, while it does not seem to affect anxiety-like behaviours in Wistar, in Kyoto, the anxiety profile was further heightened.

### Open-field (OF)

**Figure 2:**
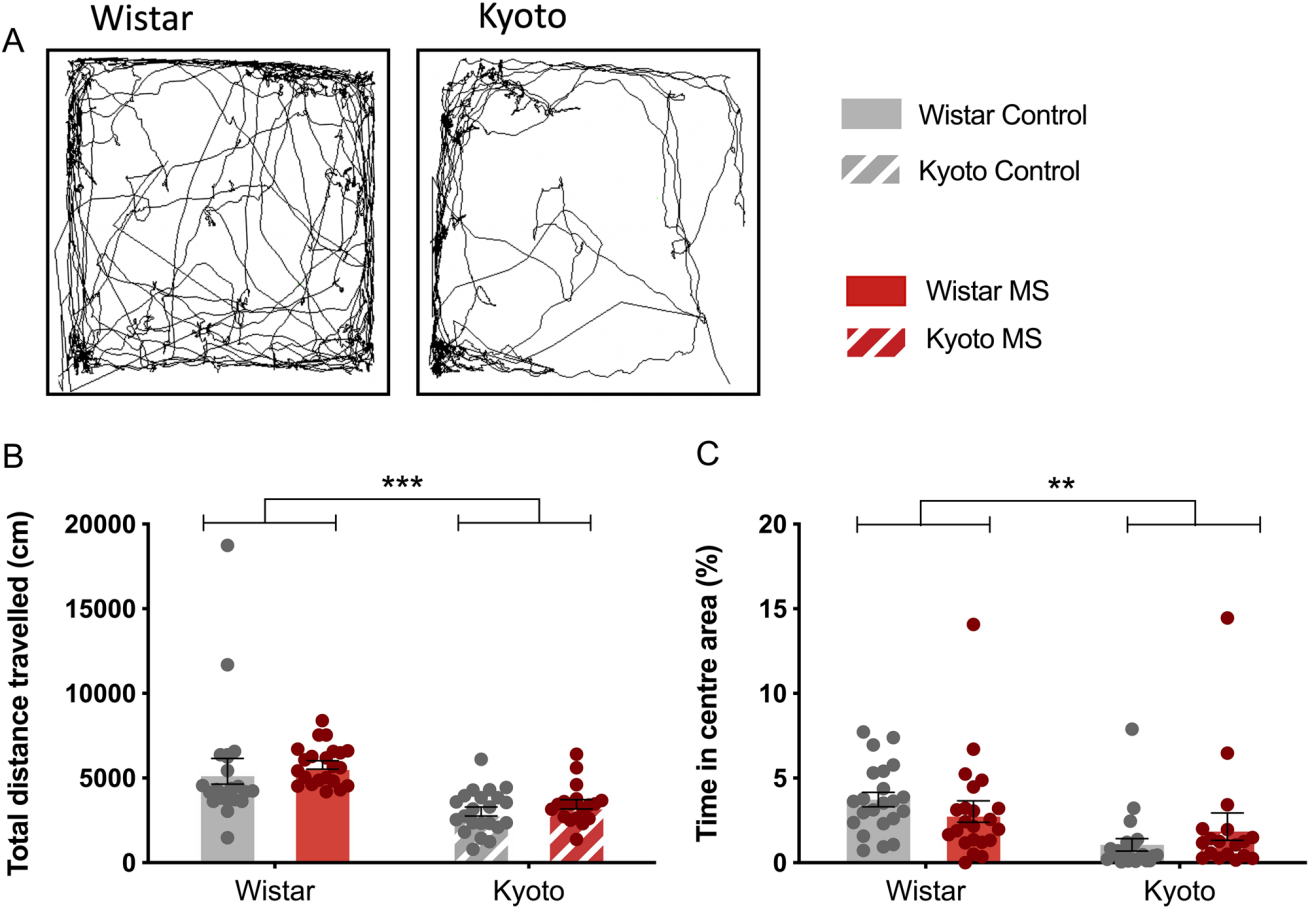
Effect of MS on the adolescent offspring locomotion in Open-Field. (**A**) example of Wistar and Kyoto control groups tracking (Smart v3.0, Panlab, Barcelona, Spain; https://www.panlab.com/en/); (**B**) total distance travelled (cm); (**C**) time in the centre area (%). Data were analysed by two-way ANOVA with Bonferroni comparisons. (**A**) data were ln transformed. *n* = 22 per group except for MS Kyoto (*n* = 18). Results are expressed as mean ± SEM. *p < 0.05, **p < 0.01, ***p < 0.001.

Next, to further corroborate the differences observed in the EPM, we performed an OF test. The Kyoto strain showed less activity in comparison with the Wistar strain again, as indicated by the total distance travelled (*F* (1,80) = 39.8, *p* < 0.001). (Fig. 2A,B). In addition, Kyoto spent less time in the centre area than Wistar [*F* (1,80) = 9.93, *p* = 0.002)], which may indicate increased anxiety in the Kyoto rats. The ANOVA also showed a significant effect of MS (*F* (1,80) = 4.15, *p* = 0.045), however, post hoc comparisons within each strain did not reveal a significant difference attributable to MS (Fig. 2C). Collectively, both EPM and OF data confirm that the Kyoto strain displays reduced activity independently of MS.

### Novel object recognition (NOR)

**Figure 3:**
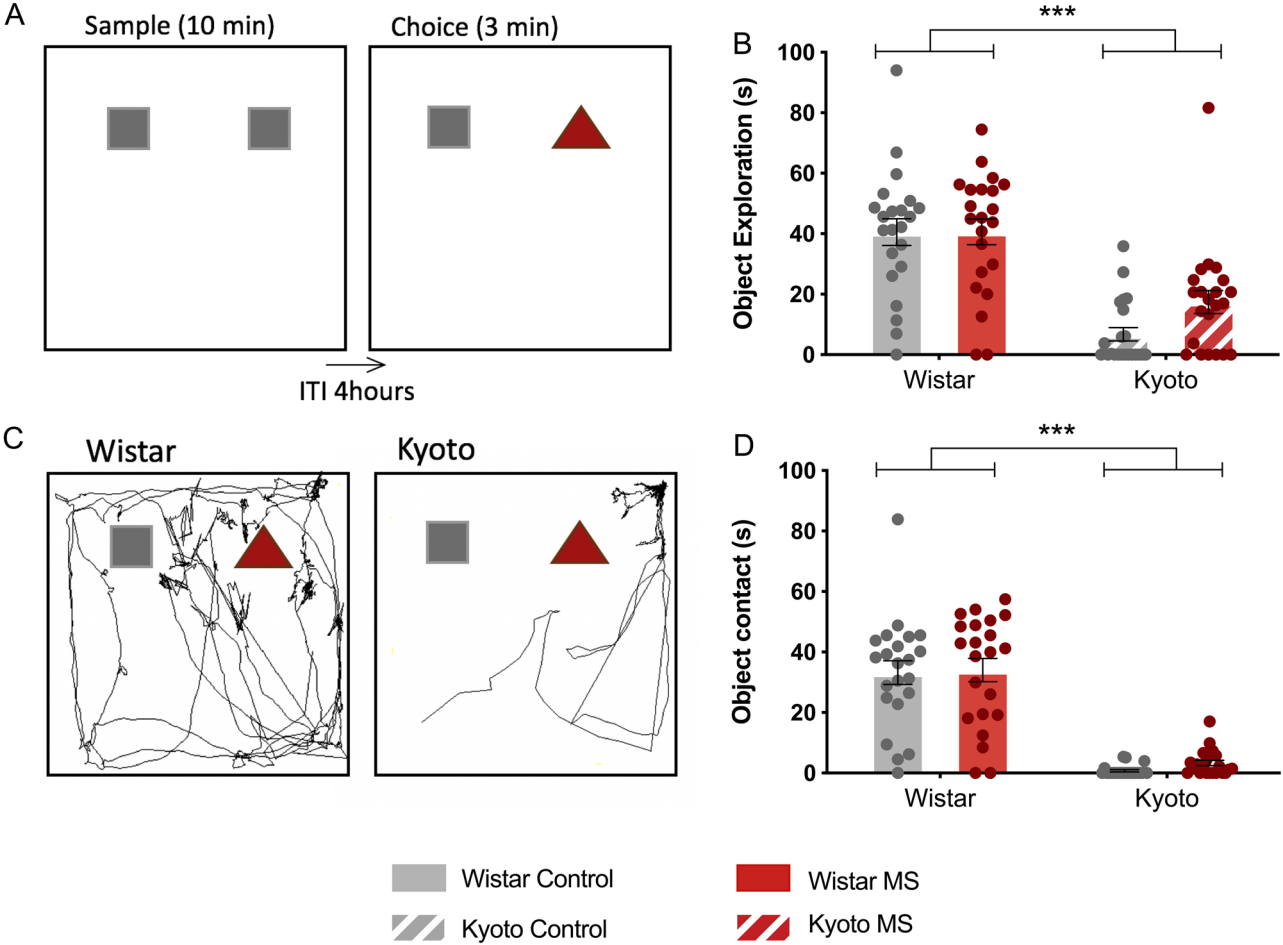
Effect of MS on the adolescent offspring memory in NOR. (**A**) schematic representation of the test; (**B**) object exploration time (s); (**C**) example of Wistar and Kyoto controls tracking (Smart v3.0, Panlab, Barcelona, Spain; https://www.panlab.com/en/); (**D**) object contact (s). (**B**) Data were analysed by two-way ANOVA with Bonferroni comparisons. (**D**) Data were analysed by chi-square. *n* = 22 per group. Results are expressed as mean ± SEM. *p < 0.05, **p < 0.01, ***p < 0.001.

Because MS was reported to induce attention and memory impairments^18^, we also evaluated how MS affected both strains when performing the NOR test (Fig. 3A). Data analysis showed that only 12 out of the 44 (27%) Kyoto rats tested explored both objects for more than 20 s in total. Moreover, Kyoto total exploration time (time exploring both objects) was lower than Wistar time (*F* (1,84) = 56.5, *p* < 0.001) (Fig. 3B,C). Our results suggest that Kyoto rats exhibited avoidance behaviour when facing novelty, as they presented significantly reduced contact with the objects during the test, when compared with the Wistar (χ^2^(1) = 27.2, *p* < 0.001) (Fig. 3D). As a consequence of the reduced exploratory behaviour displayed by the Kyoto strain, the recognition index could only be analysed for the Wistar rats. The recognition index was calculated accounting all animals that explored both objects for longer than 20 s in total (37 of the 44 Wistar rats or 84%). Wistar rats showed preference for the novel object but no differences were observed between the MS group and the control group (recognition index: 0.657 ± 0.039, 0.683 ± 0.028 respectively; *t* (35) = 0.532, *p* = 0.598), indicating that, MS did not significantly impact recognition memory in Wistar rats.

### Conditioned place preference (CPP)

**Figure 4:**
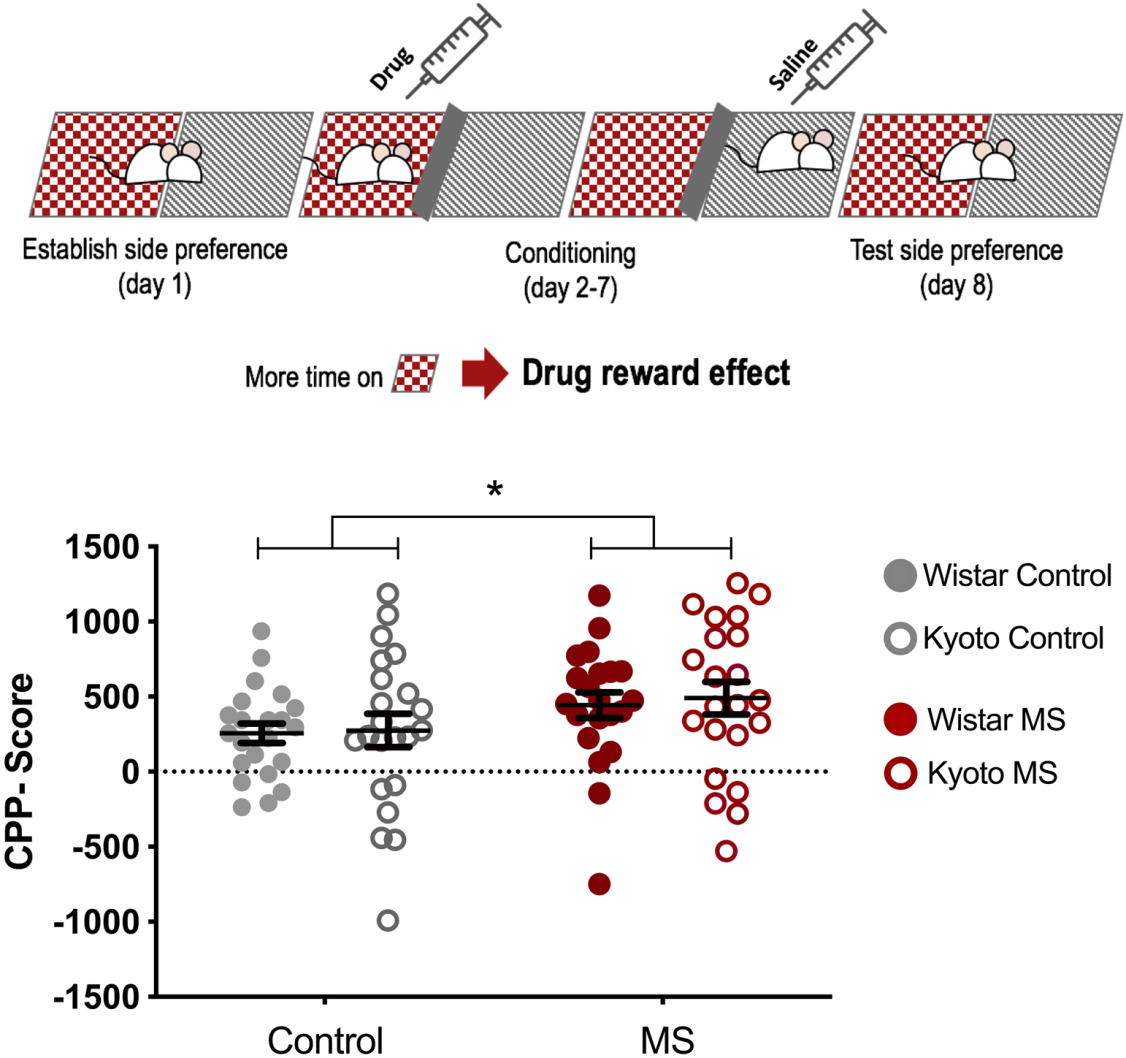
Effect of MS on the adolescent offspring vulnerability to drug abuse evaluated by CPP. CPP-score = [(time in cocaine compartment − time in saline compartment)_post-conditioning_ − (time in cocaine compartment − time in saline compartment)_pre-conditioning_]. Data were analysed by two-way ANOVA. *n* = 22 per group. Results are expressed as mean ± SEM. *p < 0.05, **p < 0.01, ***p < 0.001.

We aimed at clarifying the impact of early life stress, such as MS, in promoting vulnerability to drug addiction in adolescent rats with different predisposition to depression, therefore we used the CPP test to evaluate their behaviour under cocaine administration. The CPP results showed that all the tested animals, both Kyoto and Wistar rats were conditioned to cocaine. Moreover, results showed that MS induces heightened responses to the reward effects of cocaine (CPP-score) in both strains (MS: *F* (1,84) = 4.55, *p* = 0.036) (Fig. 4). Taking into consideration the different activity levels observed in the OF and EPM, it was hypothesized that CPP results for the Kyoto strain could be tainted by freezing behaviour. As such, an effort to evaluate the putative impact of different activity levels in drug conditioning was made and the “time in the centre area at the post-conditioning day” was analysed for strain effect, still, no strain differences were found (*F* (1,84) = 2.06, *p* = 0.155).

## Discussion

Using a validated animal model of depression (Kyoto) and the Wistar rat, we found that MS during the early days of life increases vulnerability to drug abuse in adolescent rats, independently of the strain. The results showed that MS led to an increase in the preference for the cocaine conditioned compartment by adolescent rats. To the best of our knowledge, this was the first time that adolescent rats subjected to stress during early life due to MS, were shown more prone to develop drug abuse, independently of the intrinsic genetic susceptibility for anxiety and depression. Therefore, our results support early life adversity as a critical non-genetic risk factor for the development of drug-related problems during adolescence. These findings add relevant data to the, so far, scarce literature suggesting that MS causes alterations that influence vulnerability to drug abuse in adolescent rodents^19–21^. In previous studies, also using CPP, Ganguly and colleagues^19^ (MS for 4 h daily, PND 2–20) and Viola and colleagues^21^ (MS for 3 h daily, PND 2–15) showed that MS in adolescent male rodents, but not in female, lead to a preference for the drug paired compartment. Other authors, using the maternal deprivation paradigm (MS for 24 h, PND12/13) showed increased anxiety in adolescents, but with impaired motivation for cocaine^20^. In this background, our results highlight the relevance of early life as a sensitive period for the development of differential susceptibility to drug abuse during adolescence^22^.

Adolescence is a key factor in drug use disorders^23^. In fact, adolescence is characterized by the enhancement of sensation seeking, reckless behaviour, and increased risk-taking^24^. Unveiling and understanding the risk factors associated with drug use is fundamental to develop an adequate intervention in key developmental periods. The impact of MS in adult vulnerability to drug use/abuse has already been shown for several drugs, namely opioids, ethanol, cannabinoids, methamphetamine, and cocaine^21,25–29^. As an example, studies of self-administered cocaine using rats reported that MS (separation periods of 1–6 h) potentiates cocaine-seeking behaviour^30^, leading to an increase of cocaine acquisition^31^, and continued use^32^. Simultaneously, anxiety and depression have also been previously shown to be associated with drug use and abuse^33–35^. Studies using Kyoto rats to explore the impact of anxiety and depression on drug abuse vulnerability reported that the Kyoto rat strain showed high levels of alcohol consumption^36,37^ and preference for cocaine (in doses ≤ 10 mg/kg)^38^. Along the same line, Pelloux and colleagues^39^ showed that selected anxious-Wistar rats displayed a higher preference for cocaine than non-anxious rats in the CPP test. Our results, however, show that both Kyoto and Wistar rats, if not exposed to MS, displayed a similar response to cocaine in the CPP, suggesting that the anxiety-like profile that characterizes the Kyoto strain (trait) does not seem to be a major factor for cocaine-conditioning (using a 10 mg/kg dose). Instead, our study advocates that early life stress (in this case MS) may significantly impact drug abuse vulnerability during adolescence**.** This does not exclude, however, that different doses could eventually reveal MS and strain interactions since Kyoto have already been shown to display reduced conditioning for lower doses^38^.

Although higher anxiety is frequently associated with drug addiction^40,41^, its causal role in this disorder is unclear^35,42^. Robinson and Berridge^43^ suggested that both stress and drug-induced activation of the HPA axis allow the sensitization of the mesocorticolimbic reward pathway by glucocorticoids, and this was a key factor in the transition from drug use to drug abuse. Moreover, when the transition from casual drug use to drug abuse occurs, it is usually accompanied by a shift in drug-taking motivation that departs from the reinforcing properties of drug use and ends as a way to mitigate the stress caused by withdrawal symptoms^24,44^. Along the above train of thought, and considering that the anxiety-related traits of the Kyoto and Wistar rats are different, the results from the EPM should be interpreted with caution. In our study, the EPM data suggest that Kyoto animals exhibited an increased-anxiety profile since they spent less time in the open arms than the Wistar. However, if we consider the total number of times the animals entered both arms, our data shows that Kyoto animals presented lower overall activity. In fact, Kyoto rats displayed markedly different responses in several behavioural tests when compared with the Wistar: in the EPM, the Kyoto exhibited a preference for the closed arms, displayed fewer rearings and head dippings and longer immobility time; in the OF, Kyoto rats travelled a shorter total distance and spent less time in the centre area; in the NOR, the majority of the Kyoto rats did not explore the objects for more than 20 s and avoided contact with the objects, which overruled an object recognition-index analysis. Collectively, the most striking behavioural difference between the Wistar and Kyoto is the reduced activity (or higher immobility) displayed by the Kyoto strain, which could also be related to increased fear. This lower activity was also reported in other studies in comparison with other strains^15,16,45,46^. Kyoto rats were further shown to exhibit neophobia^47^, a fear*-*related behaviour that translates into behaviour inhibition^48^, and which is considered a temperamental construct, enhancing the probability of developing an anxiety disorder^48^. In order to further understand the lack of mobility and exploration interest, future works should also consider testing the Kyoto for fear-related behaviour (e.g. fear conditioning tests)^49^. On note, in the literature addressing the performance of Kyoto rats in the EPM, Langen and Dost^50^, and Willner^51^ reported an anxious profile for this strain, however other authors^15,16^ showed no differences when comparing it with the Wistar rat. Taken together, these results suggest that the behavioural parameters measured in the EPM are not consistent when applied to characterize a rodent strain (trait), which is supposed to be stable over time, at least without treatment^52^. The EPM appears to be more related to state-anxiety characteristics^52^. Furthermore, Jastrzebska and colleagues^53^, reported unchanged levels of corticosterone in Kyoto animals measured under stress-free conditions.

In the present study, results concerning the impact of MS on the animals' emotional state showed that after the MS stress, Kyoto rats spent more time in closed arms (EPM) than MS Wistar. Moreover, MS Kyoto presented fewer total entries in either arms and higher immobility than the control Kyoto. These results are consistent with other studies also reporting that MS leads Kyoto rats to increase time in closed arms^46^ and decrease open arm entries^54^, suggesting that when Kyoto rats experience a stressful event such as MS, they are more likely to develop anxiety-like behaviour. It is notable that even though the Kyoto rat is a highly anxious strain, which could make the identification of an anxiogenic MS effect difficult due to a floor effect, significant differences to the control group were still found.

Despite other authors reported that MS also resulted in increased anxiety in Wistar rats^55,56^, our EPM data does not show a direct effect of MS on Wistar anxiety. However, the data show that MS altered the Wistar emotional state resulting in increased exploratory behaviours when compared with the control rats. The Wistar appear to have more flexible coping strategies than the Kyoto, which does not necessarily mean that their response is a more adaptative one^57^, as suggested by both strains similarly conditioning to cocaine at the CPP test.

In conclusion, our data suggest that an altered emotional state due to early-life stress increases the vulnerability to drug abuse, whereas trait anxiety and depressive mood do not significantly affect the CPP response. Studying MS as a model of early stress can provide important clues into the vulnerability to drug abuse and perhaps identify a crucial developmental window of opportunity for therapeutic intervention. This research also highlights the importance of differentiating trait from state characteristics as driving forces to drug abuse.

## Materials and methods

All experiments involving animals were approved by the Portuguese regulatory agency Direcção Geral de Alimentação e Veterinária (DGAV) and the animal ethics committee of IBMC-i3S. The animal facility and the people directly involved in animal experimentation were also certified by DGAV. All animal experiments considered the Russell and Burch 3R’s principle and followed the European guidelines for animal welfare (2010/63/ EU Directive). All time measurements were recorded in seconds and distances in centimetres.

### Animals and maternal separation (MS)

Timed-pregnant Wistar and Kyoto rats (nulliparous), supplied by Charles River Laboratories (Barcelona, Spain), on gestation day 16, were individually housed in a temperature (21 ± 1 °C) and humidity (60 ± 5%) controlled room on a 12-h light–dark cycle (light off at 12h00), with food and water freely available. The delivery day was designated as postnatal day (PND) 0. On PND 1, all pups were sexed, and dams and litters were randomly assigned to the control or the MS group. Control pups were only disturbed once a week during cage changes (i.e., animal facility reared). The MS pups were daily separated from their dam for 180 min from PND 2 to 14 inclusive (during the light period). The separation was performed by having dams and their pups removed from the home cage and placed in a different cage (pups were kept together while separated from their mother) and in different rooms to avoid ultrasonic communication^58^. Pups hypothermia was prevented by using a heating pad during MS period. Rats were weaned on PND 21 and they were co-housed (2–3 per cage) for the remainder of the experiment. All tests were performed starting at PND 33—early adolescence period (*n* = 11; strain × MS × sex). All behaviour tests had individuals from all litters.

### Behavioural tests

To evaluate if MS exacerbates the anxiety-like behaviour profile of the adolescent rats, the animals were subjected to the Elevated Plus Maze (EPM)^59^ and to the Open-Field (OF) tests. Then, the effects of MS on recognition memory were evaluated on the Novel Object Recognition (NOR) test^60^. Moreover, we analysed whether the adolescent Kyoto rats, due to their natural anxiety and depression related profile, had a higher predisposition to addiction in comparison with the Wistar rat strain and if MS impacted this predisposition. To do so, we employed the Conditioned Place Preference (CPP) test^61^, a standard preclinical behavioural model used to study the motivational effects of drugs. All adolescent rats performed the EPM test. Then, half the animals were assigned to the OF/NOR tests and the other half to the CPP test.

### Elevated plus maze (EPM)

The EPM was used to measure anxiety-like behaviour in all offspring at PND 33 morning, during the light period. The apparatus consisted of four connected arms (44 cm × 14 cm) in a plus-shaped structure elevated 72 cm above the floor. Two arms were an open platform (no sidewalls) and two were enclosed within 22-cm-high sidewalls. Arms were positioned so opposing arms were of the same type. Each rat was placed in the central platform (the intersection of the four arms (14 × 14 cm area) facing an open arm and left unperturbed for 5 min. Testing was conducted in the light phase of the light/dark cycle. The apparatus was cleaned between animals with neutral, odour free soap. The following behaviours were measured using the software The Observer XT v7.0, Noldus, Netherlands (https://www.noldus.com/observer): (a) open arms time; (b) closed arms time; (c) centre area time; (d) number of open arms entries; (e) immobility time; (f) total number of arm entries; (g) number of rearing; and (h) number of head dipping. All behaviour information in the EPM given as “time” was calculated as a percentage of the total test time.

### Open-field (OF)

Assigned rats were subjected to the OF test at PND 33 afternoon, during the dark period, to evaluate activity levels. The apparatus consisted of an empty rectangular arena (80 × 60 cm). One at a time, rats were placed in the centre area and left alone to spontaneously explore the arena for 10 min. The following behaviours were automatically recorded by The Smart v3.0, Panlab, Barcelona, Spain (https://www.panlab.com/en/): (a) time in centre area; (b) time immobile in the centre area; and (c) total distance travelled. All behaviour information in the OF given as “time” was calculated as a percentage of the total test time.

### Novel object recognition (NOR)

The same animals that were subjected to the OF test performed the NOR test to evaluate memory (PND 34). First, the animals were habituated to the apparatus. In the first NOR test day, the rats were exposed to two identical objects for 10 min. Four hours later, a novel object was introduced, randomly replacing one of the familiar objects, and the animal was left to freely explore for 3 min. It was calculated through the software The Observer XT v7.0, Noldus, Netherlands (https://www.noldus.com/observer) and The Smart v3.0, Panlab, Barcelona, Spain (https://www.panlab.com/en/) the following behaviours observed during the novel object phase (final phase): (a) the exploration time (i.e., time exploring both objects); (b) the time in contact with objects; and (c) the recognition index (i.e., time exploring novel object divided by the sum of time exploring novel object and time exploring familiar object). Data from animals with total exploration time (novel plus familiar object) of less than 20 s was excluded from the recognition index analysis.

### Conditioned place preference (CPP)

Vulnerability to drug abuse was assessed by the CPP test. The pairing of a drug with the non-preferred location followed by the change in location preference is used as indicative of the rewarding effects of a drug^62^. The apparatus consisted of a wide acrylic box divided into three compartments (two 33 × 33 cm side compartments and one 12 × 12 cm central compartment) connected by doors. One side compartment displayed black walls, while the other side compartment presented black and white striped walls. Both end compartments had different types of floor texture. A video camera was installed 1 m above the apparatus. In the first test day (PND 33), each animal was placed in the central compartment with open doors and allowed to freely explore both sides of the apparatus for 30 min (pre-conditioning). For each rat, the amount of time spent on each side was recorded. During the conditioning phase, the non-preferred side was paired with cocaine and the preferred side was paired with a saline solution. The tests were run in a biased manner with cocaine paired with the non-preferred side for each rat to avoid that different levels of bias across groups would unknowingly confound our results^61^. During the conditioning phase (6 days), the rats were intraperitoneally injected in the morning with saline and in the afternoon with cocaine (10 mg/kg body weight). Each training session lasted 30 min. This training schedule was used instead of training saline and drug on separate days because of the time constraint involved in doing developmental studies. The CPP training period was kept as short as possible to ensure that the entire experiment could be completed within the adolescent period^61^. In the last day (post-conditioning day), rats were individually placed in the central compartment with the doors opened and they were able to move freely to both side compartments, as already experienced during the pre-conditioning. This test was done in the middle of the day. The amount of time spent in each side compartment was recorded. The software Tiselius (www.tiselius.es) was used to determine the time in each compartment during both pre-condition and post-condition sessions.

### Statistical analyses

Normality was verified by the Shapiro–Wilk or Kolmogorov–Smirnov test. When measures did not validate the normality assumption but verified the homogeneity of variances the result was accepted assuming the robustness of the ANOVA test^63^. The assumption of homogeneity of variance was verified by Levene’s test. Previously to performing the ANOVA test, litter effect for all measures was considered in a Linear Mixed Model as a random effect and no litter significance was found. Then, comparisons between groups were done by a three-way ANOVA (2 × 2 × 2 factorial design), with *strain* (Wistar, Kyoto), *treatment* (control, MS), and *sex* (male, female) as independent factors. Since *sex* was found not significant (*p* > 0.05), it was removed, data were collapsed across sex, and a two-way ANOVA was conducted. Bonferroni correction was used to perform multiple comparisons. In the EPM, since some measures did not validate the assumption of homogeneity, the observations were square root-transformed (number of open arm entries, number of total entries, number of rearing, number of head dipping) and arcsine transformed (open and closed arm time, centre time and time in immobility). All the measures even with the transformation violated normality assumption. In addition, centre area time, time of immobility and number of total entries violated the assumption of homogeneity of variances even with data transformation. In these cases, a Generalized Linear Model with a robust estimator was conducted with the transformed values. Finally, due to the distribution of the number of open arm entries, a binary variable was created (0—zero open arm entries; 1—more than zero open arm entries) and a logistic regression test was used to determine the effect of strain and MS in open arm entry. Concerning OF, time in centre heterogeneity of variances was observed even using log and arcsine transformations; however, when four outliers were removed from the Kyoto MS group the assumption of homogeneity was verified. Total distance travelled data were log-transformed in order to observe the homogeneity assumption. Both measures (time in centre and total distance) did not validate the normality assumption. In NOR, time in object contact data could not be analysed with an ANOVA due to assumption violation caused by the excess of zero seconds of contact; thus, a binary variable was created (0-no contact; 1-contact) and the chi-square test was used to determine the effect of strain in object contact, taking into consideration that strain differences were observed in the other NOR measures. NOR time in exploration did not validate normality. Since most of the Kyoto animals did not explore both objects for more than 20 s in the NOR test, recognition index was calculated only for Wistar strain and Student’s t-test was used to assess the effect of MS on Wistar groups. Regarding CPP, in order to evaluate the effects of vulnerability to drug abuse, a CPP-score was created as the difference between post-conditioning and pre-conditioning preference indexes for the cocaine compartment. Preference index was calculated as the difference “time in cocaine compartment” minus “time in saline compartment”. A positive CPP-score corresponds to an increase of preference for the cocaine compartment suggesting higher vulnerability to drug abuse. Statistical significance is represented in this article by the following symbols: *p < 0.05, **p < 0.01, and ***p < 0.001. All data were analysed with the IBM SPSS Statistics 23 software.
